# Alteration of circulating redox balance in coronavirus disease-19-induced acute respiratory distress syndrome

**DOI:** 10.1186/s40560-023-00679-y

**Published:** 2023-07-05

**Authors:** Francesco Bellanti, Sławomir Kasperczyk, Aleksandra Kasperczyk, Michał Dobrakowski, Gabriella Pacilli, Giuseppina Vurchio, Alessandro Maddalena, Stefano Quiete, Aurelio Lo Buglio, Cristiano Capurso, Gaetano Serviddio, Gianluigi Vendemiale

**Affiliations:** 1 Department of Medical and Surgical Sciences, University of Foggia, Viale Pinto, 1, 71122 Foggia, Italy; 2 Department of Biochemistry, School of Medicine with the Division of Dentistry in Zabrze, Medical University of Silesia, 41-808 Zabrze, Poland

**Keywords:** Acute respiratory distress syndrome, COVID-19, Respiratory failure, Redox-dependent markers, Erythrocyte oxidative stress, Inflammation, Hypercoagulability

## Abstract

**Background:**

Mechanisms underpinning ARDS induced by COVID-19 are mostly immune-mediated, but need to be completely clarified. This study aimed to investigate redox balance in COVID-19 patients with ARDS, trying to recognize possible differences from typical ARDS related to the pathophysiology of severe disease.

**Methods:**

Patients affected by ARDS and positive for the SARS-CoV-2 virus (*N* = 40, COVID-19) were compared to ARDS patients negative to the molecular test (*N* = 42, No COVID-19). Circulating markers of redox balance were measured in serum and erythrocytes, and related to markers of inflammation and coagulability.

**Results:**

No differences in serum markers of oxidative damage were found between both groups, but a reduction in total antioxidant status and serum ceruloplasmin level was observed in COVID-19 rather than No COVID-19 patients. Redox balance alterations were described in erythrocytes from COVID-19 with respect to No COVID-19 group, characterized by increased lipofuscin and malondialdehyde concentration, and reduced glutathione S-transferase and glutathione reductase activity. These markers were associated with circulating indexes of respiratory disease severity (Horowitz index and alveolar-to-arterial oxygen gradient), inflammation (interleukin-6 and interleukin-10), and hypercoagulability (D-dimer) in COVID-19 patients with ARDS.

**Conclusions:**

ARDS caused by COVID-19 is sustained by impairment of redox balance, particularly in erythrocytes. This alteration is associated with the pro-inflammatory and pro-coagulant status which characterizes severe COVID-19.

**Supplementary Information:**

The online version contains supplementary material available at 10.1186/s40560-023-00679-y.

## Background

On March 2020, the World Health Organization declared the coronavirus disease-19 (COVID-19) as a worldwide pandemic. This condition recognizes Severe Acute Respiratory Syndrome Coronavirus 2 (SARS-CoV-2) as its etiological agent. Despite the spread of several new SARS-CoV-2 variants able to induce a huge spectrum of manifestations, acute respiratory failure is reported as the main clinical presentation and the primary cause of mortality [1].

Respiratory failure associated with COVID-19 can be classified as acute respiratory distress syndrome (ARDS) based on the Berlin definition [2]. Indeed, COVID-19-related respiratory manifestations occur within the first week, accompanied with bilateral opacities on chest radiographs, and severe hypoxemia which needs ventilatory support, in the absence of cardiac involvement [3]. However, when compared to typical ARDS, COVID-19-induced ARDS may present with no clinical respiratory distress despite severe hypoxemia—a condition named “silent” or “happy” hypoxemia [4]. Lung injury usually described in ARDS is the consequence of a disrupted barrier formed by alveolar epithelium and capillary endothelium, leading to vascular leakage and pulmonary edema. Nevertheless, ARDS induced by COVID-19 is also characterized by the extensive microthrombosis and angiogenesis of pulmonary vasculature, which can partially explain clinical manifestations, and whose mechanisms are mostly immune-mediated but remain to be completely clarified [5].

Redox balance is crucial to preserve the alveolar–capillary barrier, so that excessive generation of reactive species contributes to damage the epithelial–endothelial interface, resulting in huge leucocyte infiltration and injury amplification [6]. Reactive species are also involved in SARS-CoV-2 entry and fusion to host cells, as well as in the modulation of immune response, so that alteration in redox balance is hypothesized to be determinant in the pathogenesis and clinical severity of COVID-19 [7]. Even though the potential role of oxidative stress and the consequent possible favorable impact of antioxidant treatment in COVID-19 have been extensively reviewed [8], clinical studies are still limited and controversial. Pilot observations suggest that patients affected by severe COVID-19 show increased markers of oxidative injury and reduced antioxidant indicators [9, 10]. Nevertheless, another report showed no relationship between oxidative stress markers and COVID-19 severity, indicating that redox changes could not contribute to disease progression [11]. This latter suggestion is supported by clinical trials proving that antioxidants were not able to prevent or treat severe COVID-19 [12, 13]. Further research is then needed to better clarify the involvement of redox homeostasis in severe COVID-19, and to identify specific redox-dependent markers able to differentiate respiratory distress induced by COVID-19 from typical ARDS.

This study was designed to investigate several circulating markers of redox balance in COVID-19 patients presenting with acute respiratory distress, trying to recognize possible differences from ARDS from other causes which could be related to the pathophysiology of severe disease.

## Methods

### Study population and design

The study was conducted at the Department of “Medicina Interna e dell’Invecchiamento, Policlinico Riuniti” in Foggia (Italy). Consecutive patients admitted to our ward from June 2020 to December 2021, aged ≥ 18 years, and undergoing non-invasive mechanical ventilation within 48 h of meeting criteria for moderate/severe ARDS with partial pressure of arterial blood oxygen to fraction of inspired oxygen (PaO_2_/FiO_2_) ratio of 200 or less, were evaluated to be enrolled in the study. The FiO_2_ delivered with standard facemask was calculated as 40% with 5–6 l, 0.5 with 6–7 l and 0.6 with > 7 l of oxygen flow, and with non-rebreathing mask as 0.9 with 12–15 l of flow. Diagnosis of ARDS was performed according to the Berlin Definition criteria [2]. Patients with active neoplasia, pregnancy or active lactation, indication to extracorporeal membrane oxygenation or endotracheal intubation, Kelly scale ≥ 4, severe anemia, chronic alcoholism, use of drugs potentially affecting redox balance in the past 21 days, expected death in the following 24 h, or consent refusal, were excluded from the study.

Patients were then grouped according to the absence (No COVID-19) or the presence (COVID-19) of confirmed COVID-19, assessed by a positive polymerase chain reaction (PCR) test for SARS-CoV-2 from nasopharyngeal swab, according to World Health Organization interim guidance. Data related to demographics, comorbidities, and causes of ARDS in the No COVID-19 group were collected. In particular, comorbidities included asthma, chronic obstructive pulmonary disease (COPD), restrictive lung disease, chronic heart failure, arterial occlusive critical pathology (AOCP), obesity, hypertension, diabetes mellitus, obstructive sleep apnea syndrome (OSAS), liver diseases, cerebral stroke, lung lobectomy, venous thromboembolism, chronic kidney disease, and atrial fibrillation. Length of stay and in-hospital death were also recorded. A comprehensive pharmacological anamnesis was performed to collect baseline treatments.

All enrolled patients gave written informed consent to the study, which was approved by the Institutional Review Board and the Local Ethics Committee and conducted according to the Declaration of Helsinki.

### Laboratory analyses

Every enrolled patient underwent a simultaneous collection of venous blood samples to separate red blood cells and platelets, plasma, and serum, and to collect peripheral blood mononuclear cells (PBMCs), and arterial blood sample to perform arterial blood gas analysis, before starting the oxygen/mechanical ventilation therapy. All samples were stored at − 80 °C until the analyses at core dedicated laboratories.

Hemoglobin (Hb), white blood cells (WBC), platelet count, lymphocytes, D-dimer, erythrocyte sedimentation rate (ESR), ferritin, C-reactive protein (CRP) were determined in a blood sample. Neutrophils-to-lymphocytes ratio (NLR) was calculated by dividing the neutrophil’s number by lymphocyte’s number. The concentrations of serum interleukin (IL)-6, IL-10, tumor necrosis factor (TNF), and interferon-γ (IFN-γ), were also measured using a competitive chemiluminescence immunoassay (Randox Laboratories Ltd., Crumlin, UK), according to the manufacturer’s instructions, using the Randox Evidence Investigator.

Total oxidant capacity (TOC) and total antioxidant status (TAS) were determined in serum according to previously described methods [14].

Serum levels of protein thiol groups were assessed by measuring the absorbance at a wavelength of 412 nm of the yellow anion derivative 5-thio-2-nitrobenzoate, produced from the reduction of 5,5-dithio-bis-(2-nitrobenzoic acid) (DTNB) by sulfhydryl groups [14].

Lipofuscin concentration was measured both in serum and in erythrocytes according to the method of Tsuchida et al., as previously described [14].

Serum and erythrocyte levels of malondialdehyde (MDA), a product of lipid peroxidation, were measured fluorometrically as a 2-thiobarbituric acid-reactive substance (TBARS) according to the method of Ohkawa et al. with modifications [14]. Briefly, samples were mixed with 8.1% sodium dodecyl sulfate, 20% acetic acid, and 0.8% 2-thiobarbituric acid. After vortexing, samples were incubated 1 h at 95 °C, and butanol–pyridine 15:1 (v/v) was added. The mixture was shaken for 10 min and then centrifuged. The butanol–pyridine layer was measured fluorometrically at 515-nm and 522-nm excitation. TBARS values were expressed as MDA equivalents. Tetraethoxypropane was used as the standard.

Serum concentration of lipid hydroperoxides was assessed according to the method of Arab and Steghens, as previously described [14].

Serum concentration of ceruloplasmin was measured according to the method of Richterich, as previously described [14].

Superoxide dismutase (SOD) activity was measured both in serum and in erythrocytes according to the method of Oyanagui, as described [14]. Catalase (CAT) activity was measured in erythrocytes according to the method of Johannson and Borg, as previously described [14]. The activity of glutathione S-transferase (GST) was measured in erythrocytes according to the kinetic method of Habig and Jakoby, as described [14]. Glutathione peroxidase (GPx) activity was measured in erythrocytes following the kinetic method of Paglia and Valentine, as previously reported [14]. The activity of glutathione reductase (GR) was measured in erythrocytes, according to the method of Richterich, as described [14]. All the spectrophotometric and kinetic assays were performed using the multimode microplate reader Victor X3 (PerkinElmer, Waltham, MA, USA), while the fluorimetric and kinetic assays were performed using the multimode microplate reader Synergy HTX (Agilent BioTek, Santa Clara, CA, USA). Platelet aggregation was measured by adding collagen (1, 3 or 5 μg/ml to washed platelet suspensions and monitoring light transmission by a platelet aggregometer (MDM hematracer, MC Medical Co., Tokyo, Japan), as previously described [15].

### RNA isolation and quantitative real-time reverse-transcription polymerase chain reaction (qRT-PCR)

Total cellular RNA was isolated from PBMCs using the RNeasy Kit (Qiagen, Hilden, Germany) according to the manufacturer instructions. To ensure minimum in vitro impact on the activation status of the cells, we employed a modified gradient separation, as described previously [16]. RNA concentration was determined by spectrophotometer method at Nanodrop, measuring absorbance at *λ* = 260 nm. A260/A280 > 2 was evaluated to guarantee protein-free samples. cDNA was obtained using a random hexamer primer and a Super-Script III Reverse Transcriptase kit as described by the manufacturer (Invitrogen, Frederick, MD, USA). A PCR master mix containing the specific primers (SOD1: forward, TGT GGG GAA GCA TTA AAG G; reverse, CCG TGT TTT CTG GAT AGA GG; CAT: forward, GCC ATT GCC ACA GGA AAG TA; reverse, CCA ACT GGG ATG AGA GGG TA; GPx: forward, GGA GAC CTC ACC CTG TAC C; reverse, GTC ATT CAC CAT GTC CAC C; GST: forward, ACC TCC ACC GTA TAT TTG AG; reverse, TTG CCC CAG ACA GCC ATC TT; glyceraldehyde-3-phosphate dehydrogenase (GAPDH): forward, CAA GGC TGA GAA CGG GAA; reverse: GCA TCG CCC CAC TTG ATT TT) was added, along with AmpliTaq Gold DNA polymerase (Applied Biosystems, Foster City, CA, USA). Real-time quantification of mRNA was performed with a SYBR Green I assay and evaluated using an iCycler detection system (Bio-Rad Laboratories, Hercules, CA, USA).

### Statistical analysis

Data were expressed as count and percentages for qualitative values, and as mean ± standard deviation of the mean (SDM) or standard error of the mean (SEM) for quantitative variables. Gaussian distribution of the samples was evaluated by Kolmogorov–Smirnov test. The significance of differences between 2 groups (No COVID-19 *vs* COVID-19) was assessed by Student’s t-test (continuous variables) or in contingency tables by Pearson’s Chi-squared test and Fisher’s exact test (categorical variables). Linear regression models were used to analyze the association between PaO_2_/FiO_2_ or PAO_2_-PaO_2_ and laboratory parameters related to redox balance. All tests were 2-sided, and *P* values < 0.05 were considered to be statistically significant. Statistical analysis was performed with the Statistical Package for Social Sciences version 23.0 (SPSS, Inc., Chicago, IL) and the package Graph-Pad Prism 6.0 for Windows (GraphPad Software, Inc., San Diego, CA).

## Results

### Baseline characteristics

In the entire study period, a total of 131 patients with ARDS were considered for observation. Of these, 49 patients were excluded, while 82 patients were enrolled in the study and grouped according to the diagnosis of COVID-19 (No COVID-19, *N* = 42; COVID-19, *N* = 40; Additional file 1: Fig. S1). In the No COVID-19 group, 19 patients (45.2%) had bacterial ARDS, 10 (23.8%) were affected with viral ARDS, and 13 (31.0%) had culture-negative ARDS. Additional file 1: Table S1 summarizes the demographic and clinical features of the entire sample: no differences in age, sex, and associated comorbidities were reported between the two patient groups. Furthermore, length of stay and in-hospital death were similar among the two groups. Likewise, baseline pharmacotherapy was similar in both groups, as reported in Additional file 1: Table S2. Of note, no patients were treated with antioxidants during the study period.

The arterial blood gas measurements revealed severe respiratory failure both in No COVID-19 and in COVID-19 patients with acute respiratory distress syndrome, with similar baseline results in both groups (Additional file 1: Table S3).

Despite altered with respect to normal values, hemoglobin concentration, total WBC and platelet count were similar in both groups, even though the lymphocyte count was lower—and the NLR was higher—in COVID-19 rather than No COVID-19 patients (Additional file 1: Table S4). Even though over the upper limit of normal in all patients, circulating D-dimer levels were more elevated in COVID-19 than No COVID-19. Markers of systemic inflammation, such as ESR and CRP, were higher than the normal reference range in all patients, but no differences were reported between the two groups (Additional file 1: Table S4). Correspondingly, circulating levels of pro-inflammatory cytokines IL-6, TNF and IFN-γ, and of anti-inflammatory IL-10, were altered with respect to reference values but similar in COVID-19 and No COVID-19 patients (Additional file 1: Table S4).

### Diagnosis of COVID-19 and circulating markers of redox balance in patients with ARDS

**Fig. 1:**
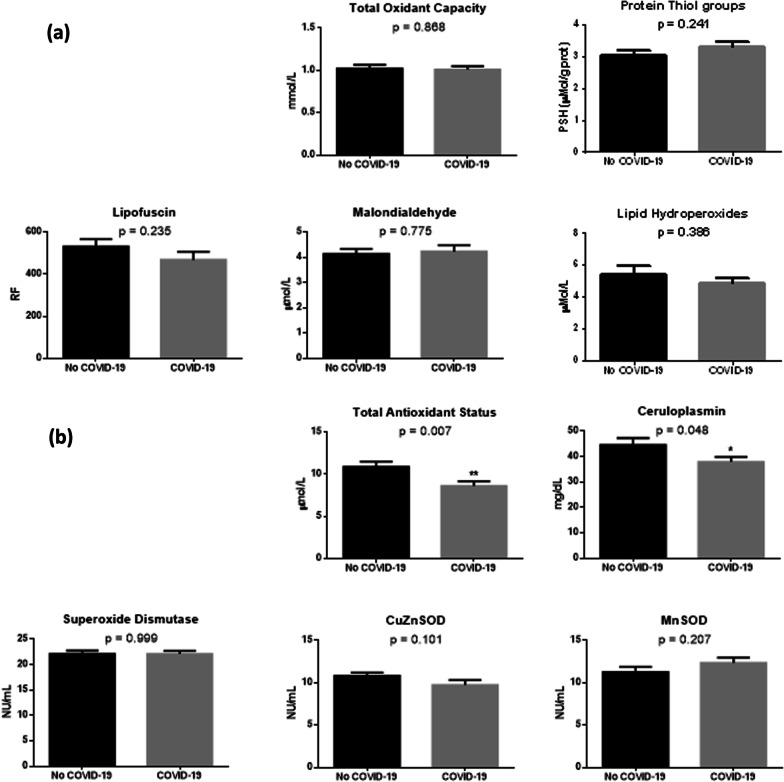
Markers of oxidative damage (**a**) and antioxidant markers (**b**) in serum of patients enrolled in the study, grouped according to the diagnosis of COVID-19. Data are expressed as mean ± standard error of the mean (SEM). Statistical differences were assessed by Student’s t-test

**Fig. 2:**
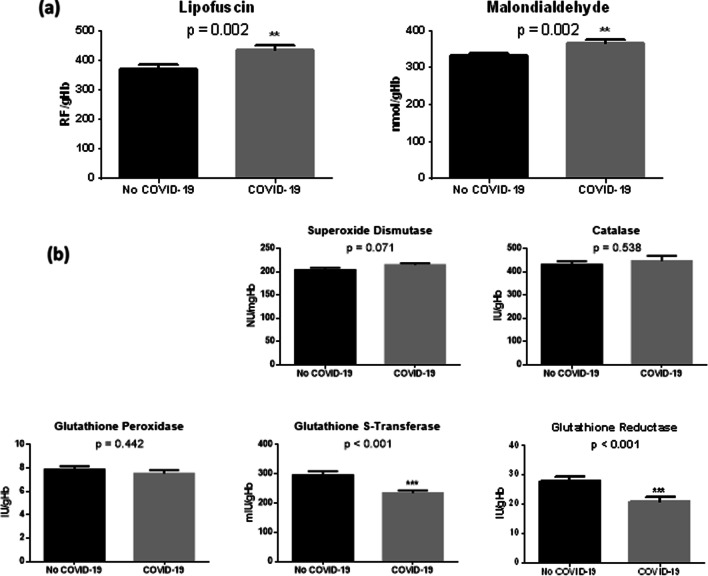
Markers of oxidative damage (**a**) and antioxidant markers (**b**) in erythrocytes from patients enrolled in the study, grouped according to the diagnosis of COVID-19. Data are expressed as mean ± standard error of the mean (SEM). Statistical differences were assessed by Student’s t-test

We tested the hypothesis whether systemic oxidative stress would be higher in ARDS patients affected by COVID-19 by measuring markers of oxidative damage and antioxidant status both in serum and in erythrocytes. Oxidative damage in serum was evaluated by measuring the TOC, protein thiol groups, lipid hydroperoxides, lipofuscin and MDA levels. As shown in Fig. 1a, no differences were reported between the two groups for any of these markers. Serum antioxidant capacity was assessed by determining the total antioxidant status, ceruloplasmin concentration, and the activity of SOD (total, MnSOD, CuSOD). Of interest, total antioxidant status and ceruloplasmin level were reduced of about 21.2% and 15.1%, respectively, in the serum of COVID-19 rather than No COVID-19 patients, while no differences in serum SOD activity were observed between the two groups (Fig. 1b). Oxidative damage in erythrocytes was determined by analyzing lipofuscin and MDA concentration. Of note, both markers were higher in COVID-19 as compared to No COVID-19 patients (Fig. 2a). To study the erythrocyte antioxidant status, the activity of SOD, CAT, GPx, GST and GR was determined. While we could not observe any difference of SOD, CAT and GPx activity between the two groups, the activity of GST and GR was lower in COVID-19 than No COVID-19 patients (Fig. 2b). Taken together, these results suggest that circulating redox balance is altered in ARDS patients and COVID-19 with respect to negative patients, defined by reduced antioxidant capacity both in serum and erythrocytes, and by increased oxidative damage in erythrocytes.

To better characterize the circulating redox profile of patients, the mRNA gene expression of antioxidant enzymes in PBMCs was quantified, and no differences were observed between the two groups (Additional file 1: Fig. S2).

### Circulating redox markers are associated with severity of respiratory failure and inflammation/fibrinolysis markers in ARDS patients with COVID-19

**Fig. 3:**
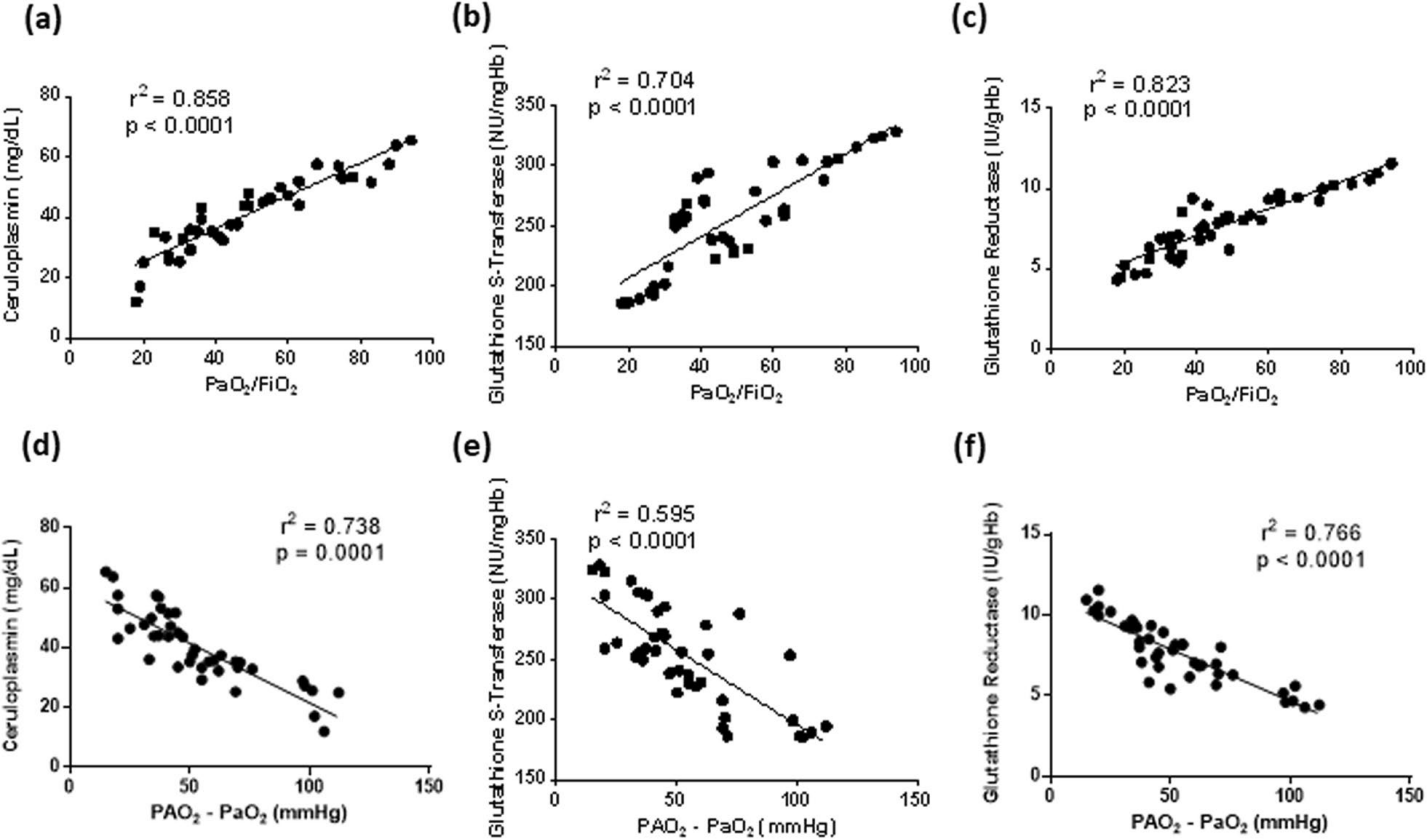
Linear regression analysis between the Horowitz index (**a-c**) or the alveolar-to-arterial oxygen gradient (**d-f**) and plasma ceruloplasmin (**a, d**), erythrocyte glutathione S-transferase (**b, e**), erythrocyte glutathione reductase (**c, f**) in patients affected by COVID-19 included in the study

To study possible associations between the severity of respiratory failure and systemic redox status in ARDS patients with COVID-19, linear regression analyses were performed between arterial blood gas measurements and altered markers of circulating redox balance. Of interest, the Horowitz index (PaO_2_/FiO_2_) was directly related to serum ceruloplasmin concentration, as well as GST and GR activity in erythrocytes (Fig. 3a–c). Similarly, the alveolar-to-arterial oxygen gradient (PAO_2_–PaO_2_) was inversely related to serum ceruloplasmin concentration, GST, and GR activity in erythrocytes (Fig. 3d–f). These data indicate that, in COVID-19 patients with ARDS, the severity of respiratory distress is dependent on the reduction of circulating antioxidant status.

**Fig. 4:**
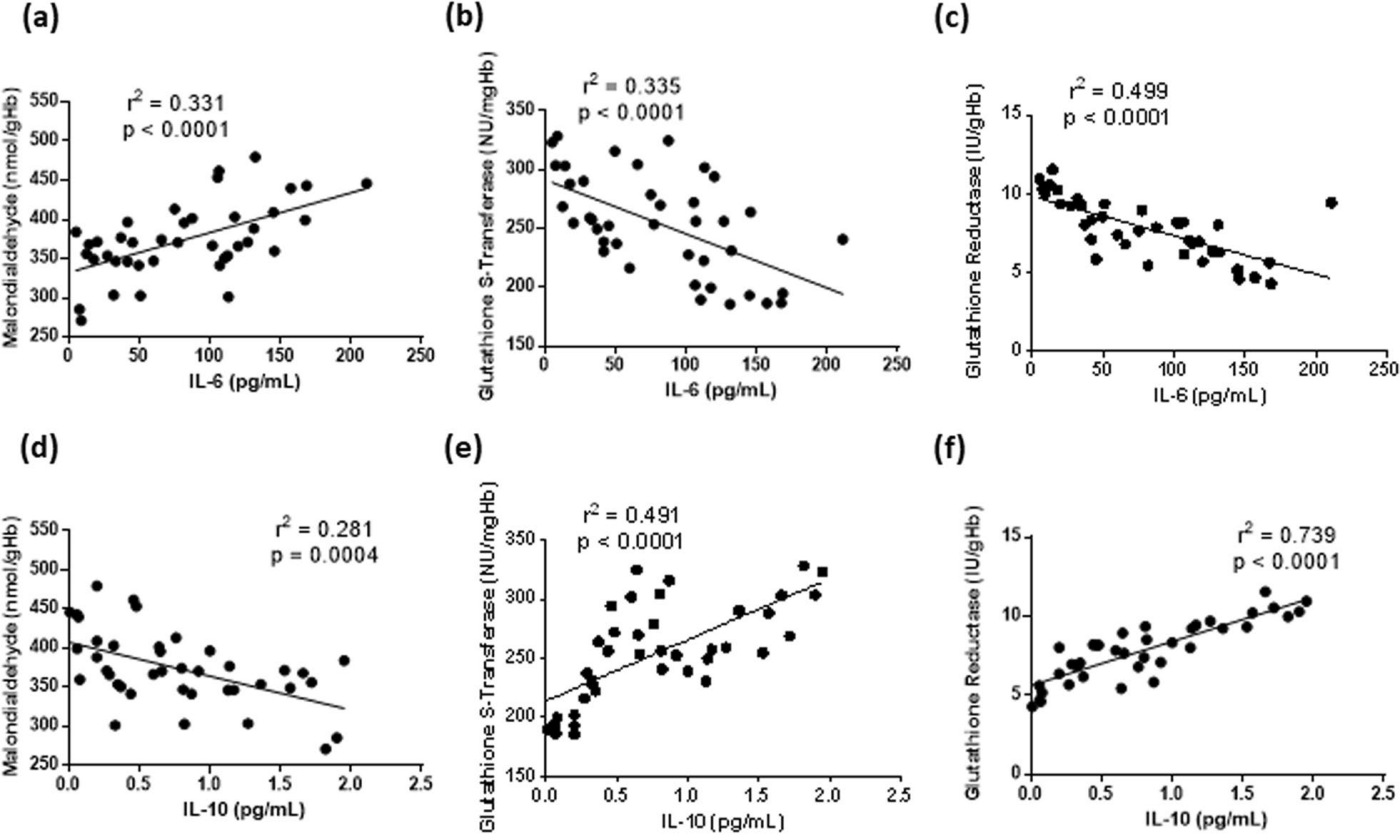
Linear regression analysis between interleukin-6 (IL-6, **a-c**) or interleukin-10 (IL-10, **d-f**) and serum malondialdehyde (**a, d**), erythrocyte glutathione S-transferase (**b, e**), erythrocyte glutathione reductase (**c, f**) in patients affected by COVID-19 included in the study

To assess the relationship between redox balance and inflammatory status, linear regression analysis between circulating markers of oxidative damage/antioxidant defense and serum cytokines were performed. Of note, erythrocyte MDA levels were directly related to IL-6, and indirectly related to IL-10 (Fig. 4a, d). We also found that GST and GR activity in erythrocytes was inversely related to IL-6, and directly related to IL-10 (Fig. 4b, c, e, f). Overall, these results suggest that the impairment in systemic redox balance directed to a pro-oxidant status can be consequent to a circulating pro-inflammatory environment in severe COVID-19.

Platelet activation is a further driving factor of COVID-19 severity [17]. Collagen-induced platelet aggregation in COVID-19 was similar to that of No COVID-19 patients (Additional file 1: Fig. S3). No relationship resulted by linear regression analysis between platelet aggregation and markers of oxidative damage/antioxidant defense.

**Fig. 5:**
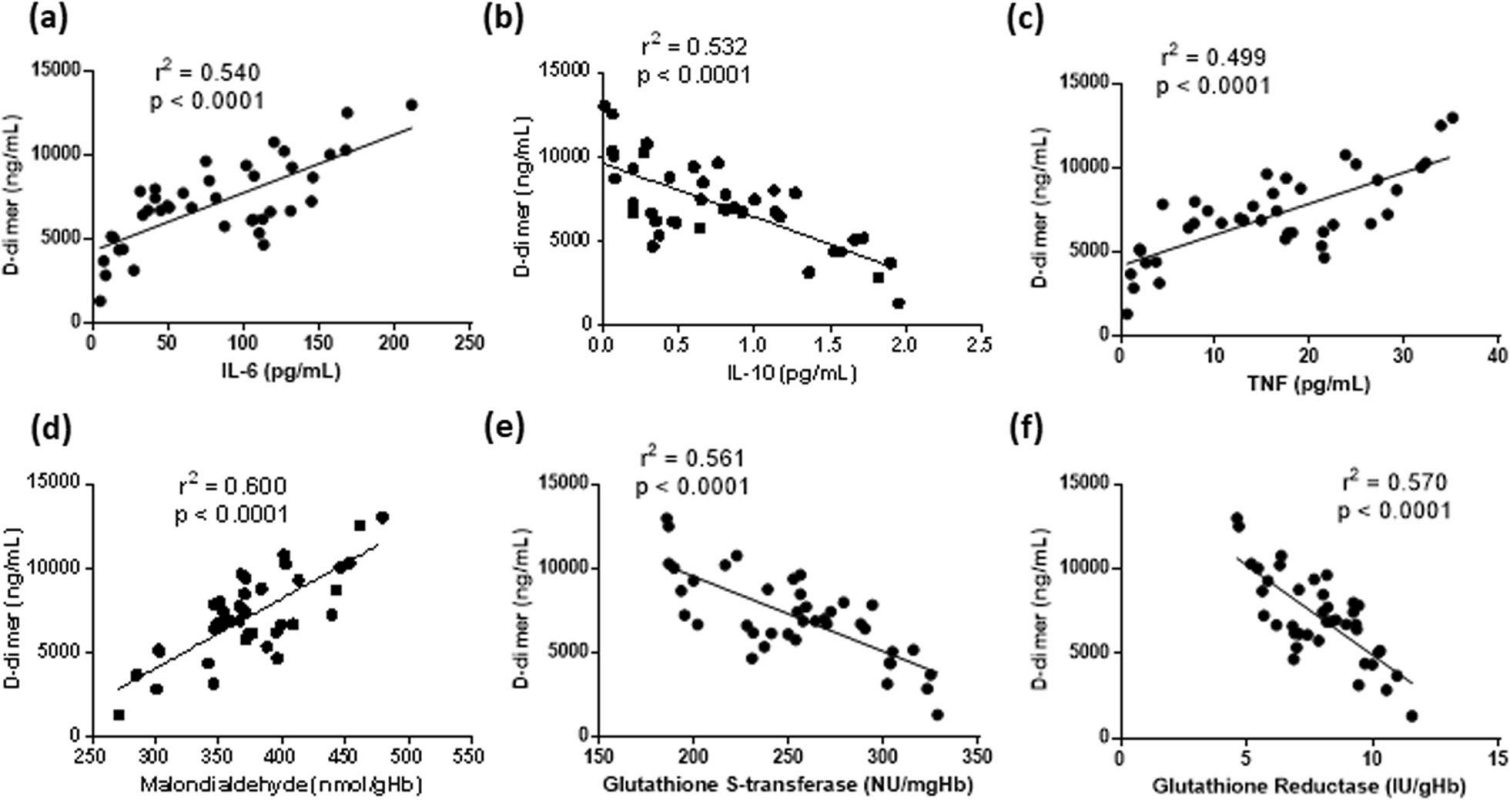
Linear regression analysis between D-dimer and circulating cytokines (**a-c**) or redox markers (**d-f**) in patients affected by COVID-19 included in the study

Spreading from formation and lysis of cross-linked fibrin, D-dimer has been suggested as a key circulating marker in COVID-19 to evaluate activation of hemostasis and to predict disease severity and unfavorable outcomes [18]. Our data show that plasma D-dimer concentration in COVID-19 patients was directly related to IL-6 and TNF, and indirectly related to IL-10 (Fig. 5a–c). Of note, we found a direct relationship between plasma D-dimer and erythrocyte MDA level, and an inverse relationship between plasma D-dimer and erythrocyte GST or GR activity in COVID-19 patients (Fig. 5d–f).

## Discussion

The present study demonstrates that, with respect to typical ARDS, respiratory distress induced by COVID-19 is associated with differences in circulating markers of redox balance. In particular, this investigation details that the impact of COVID-19 was substantial on serum antioxidant markers and on erythrocyte redox balance.

ARDS is a life-threatening disease associated with a mortality rate that ranges from 27 to 45% [2]. Even though several studies were performed to clarify the mechanism of ARDS, no effective pharmacologic therapies to target the pathophysiologic changes in ARDS were demonstrated. During the progression of ARDS, endothelial cells, alveolar cells, neutrophils, and macrophages produce reactive species that induce oxidative stress [19]. Excessive oxidant compounds can perpetuate the alveolar–endothelial barrier damage, leading to further leukocyte recruitment, delivery of cytotoxic molecules, and pulmonary edema [20]. A further burst of reactive species may be provided by oxygen therapy after desaturation, potentially inducing the anoxia–reoxygenation injury [21]. Alterations of redox balance are also considered as main factors of SARS-CoV-2 infection and pathogenesis of COVID-19, contributing to the dysregulation of immune response and to disease severity [7]. Pathophysiology of COVID-19 is more complex than typical ARDS: while the latter results from different types of injury (i.e., shock, trauma, blood transfusions, etc.) with a simultaneous impairment of epithelium and endothelium, SARS-CoV-2 disrupts the endothelium but causes a mild epithelial damage [1]. Nevertheless, impaired lung endothelium in COVID-19 may release pro-inflammatory mediators leading to the formation of microthrombi [22].

Results from this study demonstrate that patients with severe COVID-19 present with higher circulating pro-inflammatory markers and cytokines (and lower anti-inflammatory mediators) as compared to typical ARDS. However, we were not able to detect any differences in circulating markers of oxidative stress between the two groups of ARDS patients, but total antioxidant status and ceruloplasmin (a copper-containing ferroxidase) were reduced in COVID-19. Markers of oxidative damage are elevated both in typical ARDS and in severe COVID-19 [10], thus it is conceivable that serum levels of total oxidant capacity and oxidized molecules would be comparable between both conditions. Of interest, our results show altered redox balance in erythrocytes of ARDS patients with COVID-19, with respect to typical ARDS. Traditionally considered as simple oxygen transporters, erythrocytes act as determinant interorgan communication systems to regulate redox balance [23]. Indeed, erythrocytes are continuously exposed to exogenous reactive species in the bloodstream; as a consequence, erythrocytes may suffer from oxidative damage and functional impairment. To neutralize and/or minimize the effects of reactive species, erythrocytes are provided with powerful antioxidant systems. In systemic inflammation, most reactive species released by neutrophils and macrophages are gathered by erythrocytes and neutralized by cytoplasmic antioxidant enzymes [23]. Further than exogenous compounds, the continuous slow autoxidation of hemoglobin produces methemoglobin and superoxide anion [24]. Partial oxygenation of hemoglobin causes conformational modifications of the protein, with consequent increased susceptibility to autoxidation and higher affinity for plasma membrane, limiting the efficiency of antioxidant systems [25]. The present study shows increased lipofuscin and malondialdehyde, and reduced GST and GR activity, in erythrocytes from ARDS patients affected by COVID-19, suggesting that the severity of COVID-19—with respect to typical ARDS—is associated with erythrocyte oxidative stress. Furthermore, our results indicate that this condition is not dependent on alterations in the expression of antioxidant enzymes. It is worth noting that erythrocyte oxidative stress may impair oxygen delivery, amplifying the severity of COVID-19 [26].

More than PaO_2_, the PaO_2_/FiO_2_ ratio is a commonly employed marker of hypoxemia in intensive care, especially in those patients breathing a FiO_2_ > 21%, providing information about severity of respiratory failure with regard to oxygen therapy [27]. Our data show a direct relationship between the PaO_2_/FiO_2_ ratio and antioxidant markers in ARDS patients with COVID-19: as respiratory failure worsens, redox alterations (particularly in erythrocytes) occur. Even though the PaO_2_/FiO_2_ is a valuable index of lung impairment, as described by Berlin criteria in ARDS [28], an elevated PAO_2_–PaO_2_ gradient is an excellent indicator of ventilation/perfusion (V/Q) mismatch and intra-pulmonary shunting [29]. The present results also show an inverse relationship between the PAO_2_–PaO_2_ gradient and antioxidant markers in ARDS patients with COVID-19. We postulate that, with respect to typical ARDS, the severity of respiratory failure and V/Q defects induced by COVID-19 is directly related to oxidative stress.

Cytokine storm is linked to the development and progression of severe COVID-19. Our study could not demonstrate any difference in serum levels of pro-inflammatory (IL-6, TNF, IFN-γ) and anti-inflammatory cytokines (IL-10), in line with previous data [30]. However, data related to serum IL-6 level need to be interpreted with caution, since a high variability is reported in bacterial and culture-negative ARDS [30]. Nevertheless, we observed that circulating IL-6 and IL-10 values were associated with alterations of redox balance in patients with severe COVID-19, but not in typical ARDS, supporting the hypothesis that oxidative stress enhances the inflammatory response in the progression of SARS-CoV-2 infection [8]. Pro-inflammatory cytokine delivery increases the production of pro-coagulant molecules with consequent hypercoagulable state and development of microvascular thrombosis, contributing to COVID-19 severity [31]. Considered as a reliable marker of coagulation activation, D-dimer can predict severe and fatal cases of COVID-19 with moderate accuracy [32]. According to our results, patients with severe COVID-19—with respect to typical ARDS—presented with higher D-dimer levels, which were related to both systemic pro-inflammatory and pro-oxidative status in erythrocytes. Such data strengthen the hypothesis of oxidative stress—particularly related to erythrocytes—as determinant factor in the pathogenesis of severe COVID-19, since alteration of redox balance in red blood cells is associated to cytokine storm and hypercoagulability. Thus, compounds that preserve redox homeostasis in erythrocytes—such as peroxiredoxin mimetics [33]—could be considered to treat COVID-19 patients with reduced antioxidant capacity.

This study presents several limitations that should be acknowledged. First, it is conceivable that the small sample size could not draw consistent conclusions. Second, we did not compare our measurements with control groups. Third, this study did not consider specific etiologies of ARDS in the No-COVID-19 group. In addition, due to the length of the study period, we could not determine whether any specific SARS-Cov-2 variants were associated with our results. We were also not able to describe any differences in oxygen dosage or other treatment methods between the two studied groups. Also, this study did not consider arterial oxygen content (CaO_2_) to estimate ARDS severity. While this study demonstrates a correlation between redox homeostasis impairment and COVID-19-induced ARDS, it could not establish a causal relationship. Furthermore, this investigation was performed in a single center and might have presented some bias. However, even though additional multicentric studies are required to confirm our findings, this work represents a determinant step in understanding the relationship between oxidative stress and COVID-19-induced ARDS.

## Conclusions

This study demonstrates that ARDS caused by COVID-19 is sustained by impairment of redox balance, particularly in erythrocytes. This alteration is not observed in typical ARDS and is associated with the pro-inflammatory and pro-coagulant status which characterizes severe COVID-19. Even though pilot studies and clinical trials using antioxidants and redox modifiers to prevent and treat severe SARS-CoV-2 infection are advancing, this investigation provides a solid rationale to test compounds targeted at restoring erythrocyte redox balance to counteract ARDS in COVID-19.

## Supplementary Information

**Additional file 1: Fig. S1. **Enrollment and stratification of study participants. **Fig. S2. **mRNA expression of superoxide dismutase, catalase, glutathione peroxidaseand glutathione-S-transferasein peripheral blood mononuclear cells from patients enrolled in the study, grouped according to the diagnosis of COVID-19. **Fig. S3. **Collagen-induced platelet aggregation in patients enrolled in the study, grouped according to the diagnosis of COVID-19. **Table S1.** Clinical characteristics of patients enrolled in the study, grouped according to the diagnosis of COVID-19. **Table S2.** Pharmacotherapy of patients enrolled in the study, grouped according to the diagnosis of COVID-19. **Table S3.** Baseline arterial blood gas measurements in patients enrolled in the study, grouped according to the diagnosis of COVID-19. **Table S4.** Baseline biochemical characteristics, markers of systemic inflammation and circulating cytokines in patients enrolled in the study, grouped according to the diagnosis of COVID-19.

## Data Availability

The datasets used and/or analyzed during the current study are available from the corresponding author on reasonable request.

## Abbreviations

COVID-19: Coronavirus disease-19
ARDS: Acute respiratory distress syndrome
SARS-CoV-2: Severe Acute Respiratory Syndrome Coronavirus 2
AOCP: Arterial occlusive critical pathology
OSAS: Obstructive sleep apnea syndrome
PBMCs: Peripheral blood mononuclear cells
Hb: Hemoglobin
WBC: White blood cells
ESR: Erythrocyte sedimentation rate
CRP: C-reactive protein
NLR: Neutrophil/lymphocytes ratio
IL: Interleukin
TNF: Tumor necrosis factor
IFN-γ: Interferon-γ
TOC: Total oxidant capacity
TAS: Total antioxidant status
DTNB: 5,5-Dithio-bis-(2-nitrobenzoic acid)
MDA: Malondialdehyde
TBARS: 2-Thiobarbituric acid-reactive substance
SOD: Superoxide dismutase
CAT: Catalase
GST: Glutathione S-transferase
GPx: Glutathione peroxidase
GR: Glutathione reductase
GAPDH: Glyceraldehyde-3-phosphate dehydrogenase
SDM: Standard deviation of the mean
SEM: Standard error of the mean
