# Supplementary material for: Alteration of circulating redox balance in coronavirus disease-19-induced acute respiratory distress syndrome
Source: J Intensive Care. 2023 Jul 5;11:30. doi: 10.1186/s40560-023-00679-y (PMC10320967; doi:10.1186/s40560-023-00679-y)
Supplement: Supplementary file 1 — Additional file 1: Fig. S1. Enrollment and stratification of study participants. Fig. S2. mRNA expression of superoxide dismutase, catalase, glutathione peroxidaseand glutathione-S-transferasein peripheral blood mononuclear cells from patients enrolled in the study, grouped according to the diagnosis of COVID-19. Fig. S3. Collagen-induced platelet aggregation in patients enrolled in the study, grouped according to the diagnosis of COVID-19. Table S1. Clinical characteristics of patients enrolled in the study, grouped according to the diagnosis of COVID-19. Table S2. Pharmacotherapy of patients enrolled in the study, grouped according to the diagnosis of COVID-19. Table S3. Baseline arterial blood gas measurements in patients enrolled in the study, grouped according to the diagnosis of COVID-19. Table S4. Baseline biochemical characteristics, markers of systemic inflammation and circulating cytokines in patients enrolled in the study, grouped according to the diagnosis of COVID-19. [file 40560_2023_679_MOESM1_ESM.docx]

**ALTERATION OF CIRCULATING REDOX BALANCE IN CORONAVIRUS DISEASE-19-INDUCED ACUTE RESPIRATORY DISTRESS SYNDROME**

Francesco Bellanti, Sławomir Kasperczyk, Aleksandra Kasperczyk, Michał Dobrakowski, Gabriella Pacilli, Giuseppina Vurchio, Alessandro Maddalena, Stefano Quiete, Aurelio Lo Buglio, Cristiano Capurso, Gaetano Serviddio, and Gianluigi Vendemiale

**Additional file 1: Fig. S1.** Enrollment and stratification of study participants.


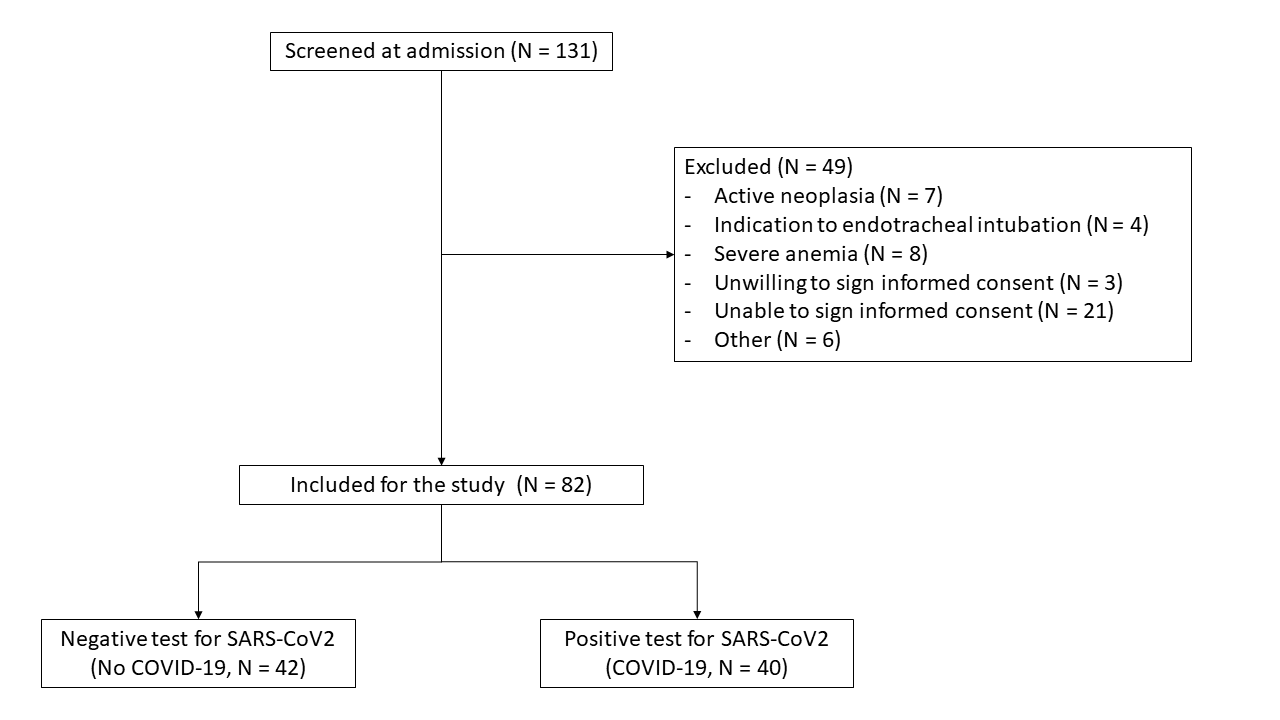


**Additional file 1: Fig. S2.** mRNA expression of superoxide dismutase (SOD), catalase (CAT), glutathione peroxidase (GSH-Px) and glutathione-S-transferase (GST) in peripheral blood mononuclear cells from patients enrolled in the study, grouped according to the diagnosis of COVID-19.


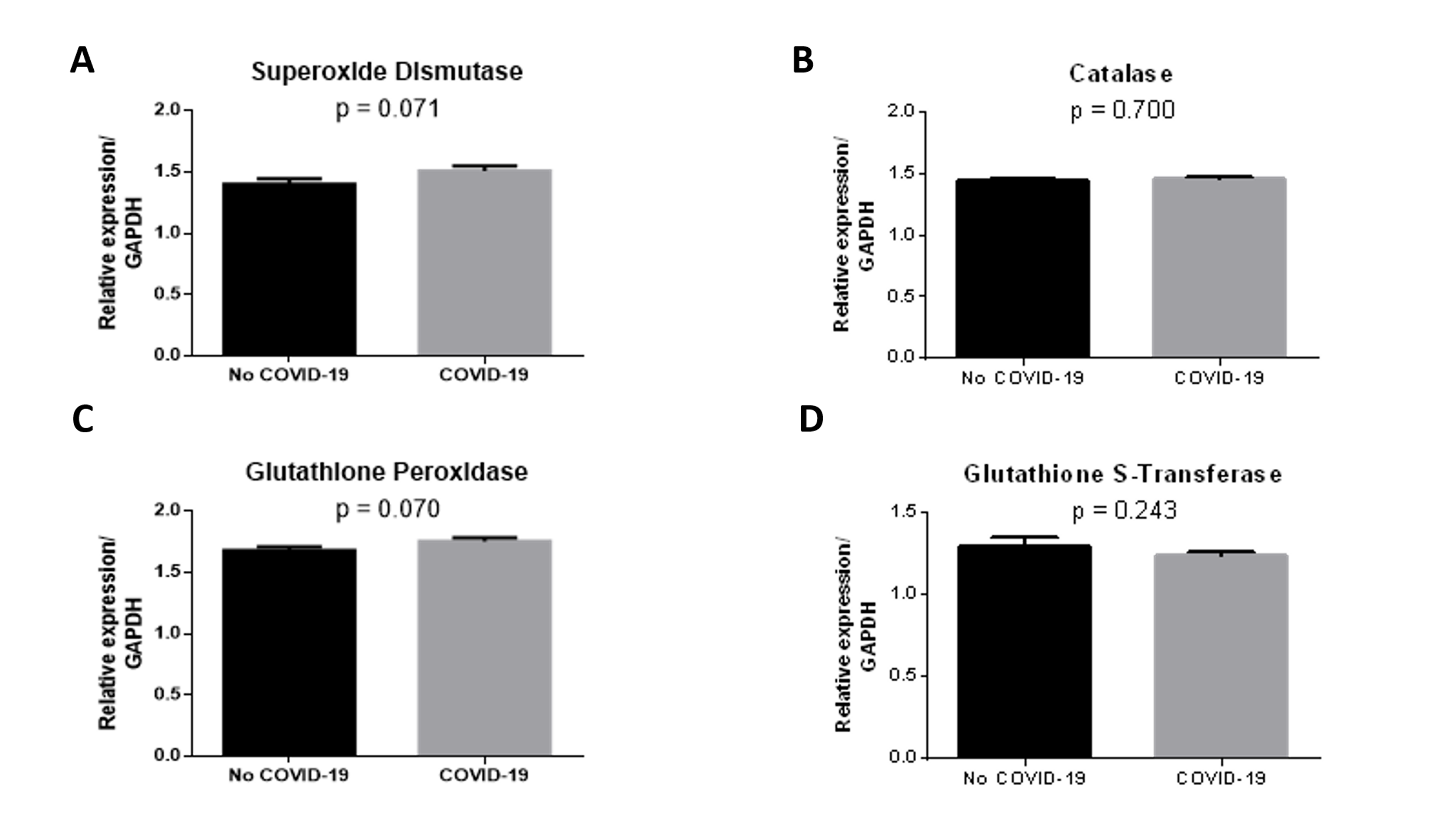


Data are expressed as mean ± standard error of the mean (SEM). Statistical differences were assessed by student’s t-test.

**Additional file 1: Fig. S3.** Collagen-induced platelet aggregation in patients enrolled in the study, grouped according to the diagnosis of COVID-19.

Data are expressed as mean ± standard error of the mean (SEM). Statistical differences were assessed by student’s t-test.

**Additional file 1: Table S1.** Clinical characteristics of patients enrolled in the study, grouped according to the diagnosis of COVID-19.

|  | No COVID-19  N = 42 | COVID-19  N = 40 | p |
| --- | --- | --- | --- |
| Age (years) | 78.5 ± 9.2 | 75.7 ± 13.6 | 0.276 |
| Sex M/F (n, %) | 14/28 (33.3/66.7) | 10/30 (25.0/75.0) | 0.407 |
| Asthma (n, %) | 0 (0.0) | 0 (0.0) | 1.000 |
| COPD (n, %) | 24 (57.1) | 16 (40.0) | 0.121 |
| Restrictive lung disease (n, %) | 10 (23.8) | 14 (35.0) | 0.266 |
| Chronic heart failure (n, %) | 18 (42.9) | 18 (45.0) | 0.845 |
| AOCP (n, %) | 4 (9.5) | 6 (15.0) | 0.449 |
| Obesity (n, %) | 22 (52.4) | 18 (45.0) | 0.504 |
| Hypertension (n, %) | 38 (90.5) | 32 (80.0) | 0.180 |
| Diabetes mellitus (n, %) | 12 (28.6) | 10 (25.0) | 0.715 |
| OSAS (n, %) | 4 (9.5) | 8 (20.0) | 0.180 |
| Liver disease (n, %) | 8 (19.0) | 10 (25.0) | 0.515 |
| Cerebral stroke (n, %) | 8 (19.0) | 4 (10.0) | 0.247 |
| Lung lobectomy (n, %) | 0 (0.0) | 0 (0.0) | 1.000 |
| Venous thromboembolism (n, %) | 6 (14.3) | 6 (15.0) | 0.927 |
| Chronic kidney disease (n, %) | 18 (42.9) | 20 (50.0) | 0.517 |
| Atrial fibrillation (n, %) | 20 (47.6) | 16 (40.0) | 0.487 |
| Length of stay (days) | 17.95 ± 12.44 | 15.73 ± 14.21 | 0.453 |
| In-hospital death (n, %) | 9 (21.4) | 12 (30.0) | 0.451 |

*COPD, chronic obstructive pulmonary disease; AOCP,* arterial occlusive critical pathology; OSAS, obstructive sleep apnea syndrome.

**Additional file 1: Table S2.** Pharmacotherapy of patients enrolled in the study, grouped according to the diagnosis of COVID-19.

|  | No COVID-19  N = 42 | COVID-19  N = 40 | p |
| --- | --- | --- | --- |
| HMG-CoA reductase inhibitors (n, %) | 18 (42.9) | 18 (45.0) | 0.845 |
| Acetylsalicylic acid (n, %) | 18 (42.9) | 16 (40.0) | 0.793 |
| ACE-inhibitors (n, %) | 24 (57.1) | 16 (40.0) | 0.121 |
| RAAS inhibitors (n, %) | 8 (19.0) | 10 (25.0) | 0.515 |
| Calcium channel blockers (n, %) | 14 (33.3) | 16 (40.0) | 0.531 |
| Beta-blockers (n, %) | 26 (61.9) | 22 (55.0) | 0.526 |
| Antidiabetics (n, %) | 8 (19.0) | 6 (15.0) | 0.626 |
| DOAC (n, %) | 18 (42.9) | 12 (30.0) | 0.227 |
| Proton pump inhibitors (n, %) | 22 (52.4) | 28 (70.0) | 0.102 |
| Corticosteroids (n, %) | 8 (19.0) | 10 (25.0) | 0.515 |
| Nitroderivatives (n, %) | 2 (4.8) | 4 (10.0) | 0.363 |

*HMG-CoA, hydroxymethylglutaryl-coenzyme A; ACE, angiotensin converting enzyme; RAAS, renin angiotensin aldosteron system; DOACs, direct oral anticoagulants.*

**Additional file 1: Table S3.** Baseline arterial blood gas measurements in patients enrolled in the study, grouped according to the diagnosis of COVID-19.

|  | No COVID-19  N = 42 | COVID-19  N = 40 | p |
| --- | --- | --- | --- |
| PaO_2_ (mmHg) | 41.0 ± 12.2 | 43.4 ± 14.3 | 0.415 |
| FiO_2_ | 0.60 ± 0.16 | 0.56 ± 0.12 | 0.206 |
| PaO_2_/FiO_2_ | 49.9 ± 18.4 | 47.7 ± 20.7 | 0.612 |
| pH | 7.44 ± 0.07 | 7.44 ± 0.05 | 0.999 |
| PaCO_2_ (mmHg) | 46.2 ± 12.0 | 46.2 ± 12.7 | 0.999 |
| HCO_3_^-^ (mmol/L) | 30.2 ± 9.8 | 28.3 ± 14.4 | 0.485 |
| PAO_2_ - PaO_2_ (mmHg) | 59.6 ± 26.8 | 55.9 ± 24.4 | 0.516 |
| Hb (g/dL) | 11.1 ± 4.7 | 10.4 ± 4.3 | 0.484 |
|  |  |  |  |
| Correlation coefficient | **FiO_2_** | |  |
| Oxygen flow rate | 0.703 | 0.796 |  |

**Additional file 1: Table S4.** Baseline biochemical characteristics, markers of systemic inflammation and circulating cytokines in patients enrolled in the study, grouped according to the diagnosis of COVID-19.

|  | No COVID-19  N = 42 | COVID-19  N = 40 | p |
| --- | --- | --- | --- |
| Hb (g/dL) | 11.3 ± 2.2 | 10.8 ± 1.3 | 0.217 |
| WBC (n/mm^3^) | 14508 ± 8840 | 13447 ± 6944 | 0.549 |
| Platelets (n*10^3^/mm^3^) | 218.4 ± 82.6 | 201.9 ± 89.5 | 0.388 |
| Lymphocytes (n/mm^3^) | 1502 ± 878 | 744 ± 435 | **< 0.001** |
| D-dimer (ng/mL) | 4902 ± 2551 | 6461 ± 2897 | **0.011** |
| NLR | 6.2 ± 4.0 | 9.6 ± 3.7 | **< 0.001** |
| ESR (mm/h) | 50.2 ± 29.8 | 68.3 ± 24.4 | 0.183 |
| CRP (ng/mL) | 97.5 ± 48.6 | 109.5 ± 94.4 | 0.468 |
| IL-6 (pg/mL) | 61.1 ± 58.7 | 80.4 ± 64.3 | 0.159 |
| IL-10 (pg/mL) | 0.79 ± 1.87 | 0.69 ± 0.84 | 0.758 |
| TNF (pg/mL) | 11.8 ± 6.87 | 13.2 ± 10.7 | 0.481 |
| IFN-γ (pg(mL) | 2.95 ± 1.78 | 3.28 ± 2.93 | 0.537 |

*Hb, hemoglobin; WBC, white blood cells; NLR, neutrophils-to-lymphocytes ratio; ESR, erythrocyte sedimentation rate; CRP, C-reactive protein; IL, interleukin; TNF, tumor necrosis factor; IFN-γ, interferon-γ.*
